# Monotropein: A comprehensive review of biosynthesis, physicochemical properties, pharmacokinetics, and pharmacology

**DOI:** 10.3389/fphar.2023.1109940

**Published:** 2023-03-02

**Authors:** Mingquan Wu, Huabing Lai, Wei Peng, Xu Zhou, Liyang Zhu, He Tu, Kezhu Yuan, Zhirui Yang

**Affiliations:** ^1^ Department of Pharmacy, Sichuan Orthopedic Hospital, Chengdu, Sichuan, China; ^2^ Department of Rehabilitation and Prosthetic Orthopedics Center, Sichuan Orthopedic Hospital, Chengdu, Sichuan, China; ^3^ Department of Scientific Research, Sichuan Orthopedic Hospital, Chengdu, Sichuan, China; ^4^ Department of Nuclear Medicine, Chengdu Second People’s Hospital, Chengdu, Sichuan, China

**Keywords:** monotropein, biosynthesis, physicochemical properties, pharmacokinetics, pharmacology

## Abstract

Monotropein, a principal natural compound in iridoid glycosides extracted from *Morindae officinalis* radix, has potent pharmacological activities. To understand and utilize monotropein, we systematically summarized the studies on monotropein, including its biosynthetic pathway, physicochemical properties, pharmacokinetics, and pharmacology. Interestingly, we found that the multiple bioactivities of monotropein, such as anti-osteoporosis, anti-inflammation, anti-oxidation, anti-nociception, and hepatic or renal protection, are closely associated with its capability of downregulating the nuclear factor-κB signaling pathway, inhibiting the mitogen-activated protein kinase signaling pathway, attenuating the activation of nuclear factor E2-related factor 2/heme oxygenase-1 signaling pathway, and regulating the mammalian target of rapamycin/autophagy signaling pathway. However, the clinically therapeutic effects and the potential problems need to be addressed. This review highlights the current research progress on monotropein, which provides a reference for further investigation of monotropein.

## 1 Introduction

Traditional Chinese herbal medicine has been verified and applied in long-term clinical practice and has become a major source of diverse potential medical products characterized by their potent biological activities and minor side effects. Monotropein, extracted from *Morinda officinalis* How, *Pyrola calliantha* H. Andres or *Pyrola decorata* H. Andres (Wen et al., 2019; Yu et al., 2021), is the most abundant constituent in iridoid glycosides found in *Morindae officinalis* radix (about 13 mg/g) (Zhang et al., 2017). According to the theory of traditional Chinese medicine, *M. officinalis* radix is used to improve the syndrome of kidney Yang deficiency, wherein monotropein is the main component for “reinforcing kidney to strengthen Yang” (Zhang C. et al., 2022) and has pharmacological activities that are responsible for the therapeutic effects in patent medicine (Wang et al., 2019; Choi et al., 2020). Reportedly, monotropein is a critical marker compound in plant affinity, specificity of secondary metabolite accumulation, traditional efficacy correlation, blood component, processing correlation, and component measurability, as well as the core element contributing to intervening diseases and controlling quality transfer (Huang et al., 2022). To reveal its potential in drug development, this review systematically summarizes the studies on monotropein with respect to its biosynthetic pathway, physicochemical properties, pharmacokinetics, pharmacological effects, and molecular mechanisms to provide comprehensive information for further in-depth studies on monotropein.

## 2 Biosynthesis, extraction and isolation, physicochemical properties, pharmacokinetics, and pharmacological activities of monotropein

### 2.1 Biosynthesis

**FIGURE 1:**
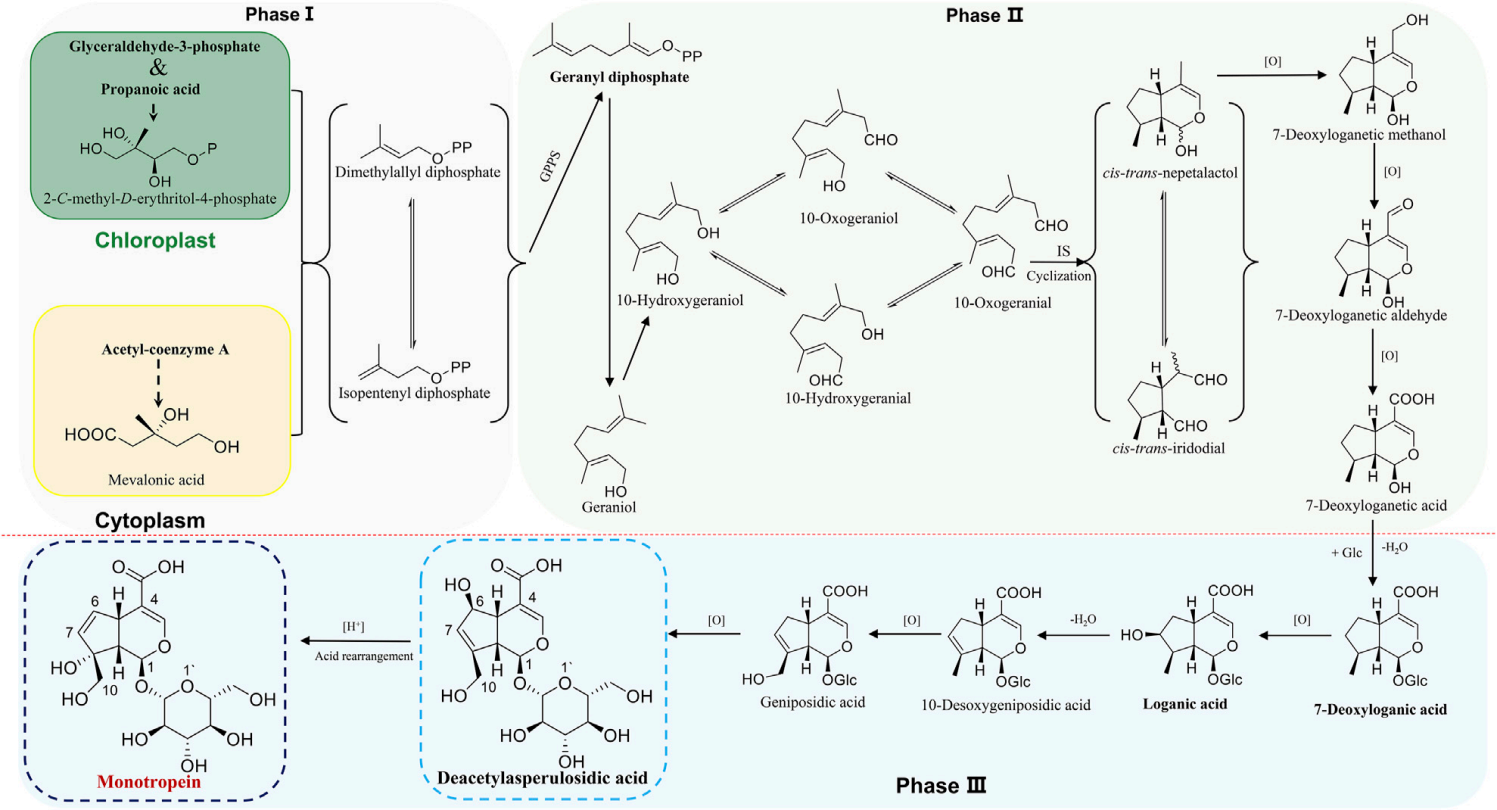
The proposed biosynthesis of monotropein.

The iridoids and iridoid glycosides belong to cyclic monoterpene derivatives with cyclopentane as the structural unit, including two types of iridoid and secoiridoid. As the basic nucleus of iridoids, iridoid consists of cyclic alkenyl ethers and alcoholic hydroxyl group in C_1_, which makes this semiacetal hydroxyl an active site, linking to 1–3 glucoses as glycosides in medicinal plants. The biosynthesis of monotropein is the conversion of a simple natural product into a complex desired product in an organism under enzymatic conditions. It is effectuated in three phases as follows: Firstly, the intermediates of isopentenyl diphosphate (IPP) and dimethylallyl diphosphate (DMAPP) are generated in phase Ⅰ, which is the origin of phytochemistry. Subsequently, geranyl diphosphate (GPP) is generated based on the condensation of IPP or DMAPP in a head-tail or head-head manner under geranyl pyrophosphate synthase (GPPS), a demarcation point of producing various terpenes and alkaloids, including iridoids and iridoid glycosides. Notably, IPP and DMAPP are generated from mevalonic acid (MVA) biosynthetic pathway in the cytoplasm and methylerythritol phosphate (MEP) pathway in chloroplasts, and the IPP and DMAPP from two sources could exchange mutually. Although the emergence of the MVA pathway plays a key role in the evolution of plants that facilitates land colonization, embryo development, and adaption to new and varied environments (Pu et al., 2021), accumulating evidence supports that the MEP biosynthetic pathway is the dominant in synthesizing IPP and DMAPP to iridoids and iridoid glycosides (Hong et al., 2003; Burlat et al., 2004; Oudin et al., 2007; Zhang et al., 2012). In phase Ⅱ, the intermediate product and basic nucleus of iridoid are synthesized *via* dephosphorylation, oxidation, and cyclization in sequence. Specifically, GPP is transformed into geraniol through dephosphorylation. Furthermore, 10-hydroxygeraniol, 10-oxogeraniol (or 10-hydroxygeranial), and 10-oxogeranial are synthesized sequentially by chain oxidation. Importantly, the first cyclization is formed for cyclopentane structure unit and *cis*-*trans*-iridodial, which are the key skeletons in the phytochemistry of iridoids and iridoid glycosides and are catalyzed from 10-oxogeranial under iridoidsynthase (IS) (Geu-Flores et al., 2012). Finally, the basic iridoid of *cis*-*trans*-nepetalactol with cyclic alkenyl ethers and alcoholic hydroxyl is formed through enol tautomerism and nucleophilic addition reaction to *cis*-*trans*-iridodial in the second cyclization. Phase Ⅲ is a post-modification process, including hydroxylation, glycosylation, methylation, acylation, or binding to small molecules, wherein various secondary metabolites are synthesized based on *cis*-*trans*-nepetalactol (Wu and Liu, 2017). Typically, the proposed biosynthetic pathway of monotropein is derivatized from loganin/deacetylasperulosidic acid dynamic route (Figure 1) (De Luca et al., 2014; Miettinen et al., 2014; Salim et al., 2014; Song et al., 2014; Yang et al., 2018). Monotropein and deacetylasperulosidic acid from *M. officinalis* root are secondary metabolites and volatile at 1.27–21.78 mg/g and 3.16–5.01 mg/g, respectively, suggesting that the quality control based on the content determination of monotropein or deacetylasperulosidic acid should be improved (Zhao et al., 2018a).

### 2.2 Extraction and isolation

Monotropein is abundant in the root, stem, and leaf of *M. officinalis*, with the highest content in the leaf (about 2%). During the two to six growth periods, the content of monotropein accumulates continually (Feng et al., 2017). In addition, *M. officinalis* is distributed in the south of China. The content of monotropein in *M. officinalis* is greatly affected in different habitats. The order of content of the wild samples of *M. officinalis* is Hainan < Guangdong < Guangxi, and that in the cultivated samples is Fujian < Guangdong < Guangxi (Liu D. F. et al., 2010). As the roots of *M. officinalis* are the medicinal parts used in traditional Chinese medicine, the preparation procedure for monotropein is mainly focused on the roots.

Chemically, the structure of monotropein consists of polar groups, such as carboxyl and hydroxyl. Monotropein is soluble in ethyl acetate, n-butanol, acetone, and easily soluble in ethanol, methanol, aqueous alcohol solution, and water. *M. officinalis* contains oligosaccharides and polysaccharides, and hence, monotropein is extracted with aqueous alcohol solution. Because the water medium has a swelling effect for the medicinal materials, it is more conducive to use methanol or ethanol to penetrate and dissolve, thus improving the extraction rate of monotropein in *M. officinalis*. The yields rate could be about 2.17% by ultrasonic extraction with 80% methanol aqueous solution after the material was pulverized to a coarse powder (Xu and Ding, 2007). Moreover, a valuable reference is provided that the combination of traditional water decoction and biological enzyme technology could be introduced into the extraction of monotropein from *M. officinalis*. With the optimization of enzyme amount, the temperature during enzymolysis, the ratio of material to liquid, and the extraction time, the yield of monotropein could be 13.61 mg/g, which is much higher than the traditional water decoction extraction (4.08 mg/g) (Song et al., 2018).

The appropriate isolation is based on a high extraction rate with the advantages of energy conservation, environmental friendliness, labor protection, and low production risk. Monotropein can also be extracted using heated reflux extraction with 80% ethanol. The mixed decoction is concentrated to thickened paste with no alcohol flavor in the hypobaric environment. Then, the paste is dispersed with water and further extracted with petroleum ether, ethyl acetate, and n-butanol successively. The n-butanol-extracted delamination is collected to obtain the extract, which is redispersed with water and loaded onto the macroporous resin to enrich the iridoids and remove the impurities of polysaccharide with gradient elution of water and 30%, 60%, and 95% ethanol. The 30% ethanol elution is collected, as it contains the most abundant content of iridoids. Finally, the extracts are repeatedly separated using normal and reversed-phase column chromatography, on which highly pure monotropein could be obtained (Peng et al., 2008). Importantly, one patent was suitable for the mass preparation of monotripein. *M. officinalis* (200 g) is pulverized to powder and mixed with 2 L of 50% ethanol, followed by ultrasonic extraction twice. The decoction is mixed and condensed to thick paste that is dispersed with water and loaded onto polyamide resin after filtration. First, the deionized water is used to remove the water-soluble impurity, and 30% ethanol is used in elution until the eluent is colorless. Then, the collected ethanol elution is then concentrated and crystallized for 6 h, and 105 g crude crystal can be obtained. The crude crystal is solubilized by heat ethyl acetate and re-crystallized for 7 h. After refiltration, the crystal is dried at a low temperature. This approach of isolation retrieved 0.17 g monotropein, with 97.4% purity (quantified using high-performance liquid chromatography) and 0.0828% yield (mass ratio of obtained pure monotropein to the raw plant of *M. officinalis*). High efficiency can be achieved because ultrasonic wave promotes the penetration of solvent into the plant, with low energy consumption and cost as opposed to heated reflux extraction. The use of 30% ethanol ensures less toxicity compared with methanol. Finally, usage of ethyl acetate as the crystallization solvent reduces the production risk, and recrystallization can guarantee high purity of monotripein, wherein the complexity of repeated column chromatography and consumption of a large number of organic reagents can be avoided (Liu J. et al., 2010).

### 2.3 Physicochemical properties

**FIGURE 2:**
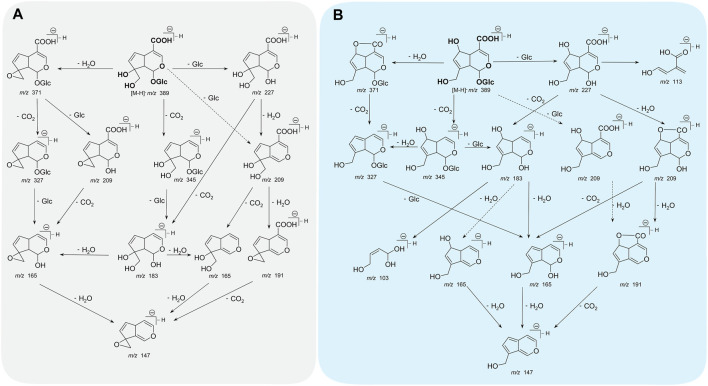
The proposed fragmentation pathway of monotropein **(A)** and deacetylasperulosidic acid **(B)** in negative ion mode of ESI-MS/MS. Solid-line arrows indicated higher probability and dotted-line arrows indicated lower probability in fragmental process.

Monotropein is a white crystalline solid or amorphous powder with bitter taste. In the color reaction of monotropein, turquoise blue, blue converted into purple, and blue-black could be observed when monotropein is treated with antimony trichloride, phosphomolybdic acid, and concentrated hydrochloric acid, respectively. Moreover, the ultraviolet (UV) and infrared (IR) absorption spectra are as follows: UV λ_max_ (MeOH) = 235 nm, logε = 3.80, and IR ν_max_ (KBr) = 3,580, 1700, 1,675, 1,645, and 1,618 cm^–1^ (Chen and Xue, 1987). Given that the identification of monotropein might be inaccurate in the microscale (mass ratio at 0.01–1%) or trace (mass ratio <0.01%) analysis, the mass spectrometry-based techniques are considered robust approaches with the advantages of high sensitivity, high selectivity, high resolution, and rapidity (Ma et al., 2021). Hence, the identification accuracy could be enhanced by interpreting the type and abundance of ion fragments as the basic data, such as acquisition rate, resolution, precursor ion mass window, fragmental voltage, or collision energy, which could be retrieved from the instrument performance and parameter settings (Kind et al., 2018). Electron spray ionization was used to obtain the mass spectra in either positive or negative ion modes, and higher sensitivity could be achieved in the negative ion mode (Zhou et al., 2018). In addition, the stability of iridoid glycosides is lower in the positive ion mode than in the negative ion mode, in which a series of garbage fragment ions could be produced easily. In contrast, abundant information of fragment ions for interpreting the structure with less interference from the background could be obtained in the negative ion mode (Li et al., 2013). The mass fragmental regularity of monotropein occurs in two stages. The neutral functional substitution groups are separated from the parent nucleus, and the main and typical losses are H_2_O (18 Da), CO_2_ (44 Da), glucose residue (Glc) (162 Da), and glucose (180 Da). Moreover, the dihydropyran and glucose rings are fractured due to hemiacetal structure isomerization. The characteristic ions formed from parent ring breakage can be used to identify the structure of the parent nucleus (Zhao et al., 2018b). The proposed fragmentation pattern of monotropein in the negative ion mode of ESI-MS/MS is summarized in Figure 2A (Heffels et al., 2017; Zhao et al., 2018b).

Monotropein is converted into deacetylasperulosidic acid in acidic artificial gastric fluids (pH = 1.41) without any enzyme but not in alkaline artificial intestinal juice (pH = 6.88) or neutral aqueous solution (Wang et al., 2011). Interestingly, a diametrically opposed result was also observed, in which monotropein was stable in simulated gastric fluid (pH = 1.2) and intestinal fluid (pH = 6.8) (Zhou et al., 2018). Regarding the acid rearrangement in biosynthetic pathway, monotropein had the potential of conversion into deacetylasperulosidic acid. And pure monotropein or that in complex extraction of major iridoid glycosides from *M. officinalis* under acid condition could be partly converted to deacetylasperulosidic acid (Zhang et al., 2017; Shen et al., 2018; Shen et al., 2019). As monotropein is usually orally administered, it is necessary to investigate the fragmentation pattern of deacetylasperulosidic acid, which could be distinguished based on retention behavior, accurate mass, fragment ions, and neutral losses and further validated using standard compounds. As the isomers of monotropein, deacetylasperulosidic acid have distinct polarity and similar chromatographic behavior and mass spectrometry information (m/z fragmentation transition of both compounds as 408 → 211), while its retention time is always <5 min in reverse column with monotropein syringed first (Shen et al., 2018). Notably, the monotropein and deacetylasperulosidic acid may not be separated under specific chromatographic conditions or samples containing excessive interfering substances (Yu et al., 2021). In addition, the signals of deacetylasperulosidic acid are similar to monotropein with respect to accurate mass, fragment ions, and neutral losses. Consequently, the fragmentation pattern (Figure 2B; Table 1) could not be explained by the structural difference in monotropein and deacetylasperulosidic acid (Wang et al., 2017; Zhao et al., 2018b; Zhao et al., 2018), which need to be elucidated by comparing the ion abundance to identify the deacetylasperulosidic acid. This acid displays the prior losses of Glu and Glc residues from principal [M-H]^–^ ion at m/z 389.1098, and an apparent fragment ion [M-H-Glc]^–^ at m/z 227.0556 was identified by a loss of a glucose residue, following a loss of a CO_2_ from [M-H-Glu]^–^ at m/z 209.0447 or [M-H-Glc]^–^ at m/z 227.0556 (Zhao et al., 2021).

**TABLE 1:** The fragmental ions of monotropein and deacetylasperulosidic acid in negative ionization mode.

Compounds	Precursor ion (m/z)	Product ion (m/z)	Solvent	References
Monotropein	389 [M-H]^−^	227, 191, 165, 147, 135, 119	Methanol and water (50:50, v/v)	Zhou et al. (2018)
Monotropein	389.0098 [M-H]^−^	227.9874, 226.9836, 164.9952, 146.9876, 118.9832	Methanol	Zhao et al. (2018b)
Monotropein	435 [M + Formate]^−^, 389 [M-H]^−^	371, 345, 327, 227, 209, 191, 183, 165, 147	Distilled water	Heffels et al. (2017)
Monotropein	389.1066 [M-H]^−^	226.9712, 190.9609, 165.0189, 164.9873, 146.9802, 146.9305	Methanol	Yu et al. (2021)
Monotropein	389.1092 [M-H]^−^	227.0547, 209.0442, 183.0649, 165.0548, 137.0604, 119.0353, 89.0256	Water	Shen et al. (2018)
Deacetylasperulosidic acid	389.0139 [M-H]^−^	345.0210, 226.9836, 208.9785, 183.0025, 164.9978, 146.9901, 118.9854, 88.9905	Methanol	Zhao et al. (2018a)
Deacetylasperulosidic acid	435 [M + Formate]^−^, 389 [M-H]^−^	371, 345, 327, 227, 209, 191, 183, 165, 147	Distilled water	Heffels et al. (2017)
Deacetylasperulosidic acid	389.1072 [M-H]^−^	227.0082, 190.9921, 165.0189, 146.9305	Methanol	Yu et al. (2021)
Deacetylasperulosidic acid	389.1089 [M-H]^−^	227.0558, 183.0658, 179.0567, 161.0455, 113.0238	Methanol	Zhao et al. (2018)
Deacetylasperulosidic acid	389.1098 [M-H]^−^	227.0556, 209.0447, 165.0551, 147.0445, 139.0389, 119.0359, 89.0258, 59.0176	Water	Zhao et al. (2021)

Monotropein can be quantified using high-performance liquid chromatography with UV detection. However, owing to the limitations of low specificity, sensitivity, and accuracy, this method is not a satisfactory option. As a common analytical method in mass spectrometry, multiple reaction monitoring (MRM) is widely recognized for its high specificity, accuracy, automatic throughput, sensitivity, reproducibility, and wide linear dynamic range. Due to less interference by other components in the samples, mass spectrometry with MRM mode overcomes the issue of poor resolution of high-performance liquid chromatography. Combined with high-performance liquid chromatography, mass spectrometry with MRM mode is recommended to quantify monotropein.

### 2.4 Pharmacokinetics

From the perspective of therapeutic or economic benefit, it is crucial to investigate the pharmacokinetic behavior of monotropein in absorption, distribution, metabolism, and excretion. Monotropein was well absorbed in the Caco-2 cell monolayer model, and the bilateral apparent permeability coefficients (P_app_) at low concentrations were significantly greater than those at medium or high concentrations. After the addition of verapamil hydrochloride, the P_app_ of monotropein changed slightly, indicating that the carrier was not required in the intestinal absorption. Also, monotropein was passively transported across the transmembrane and showed self-inhibition at the specific concentrations (Zhang D. et al., 2022). In the intestinal perfusion model, the small intestine was the main absorption site after oral administration. The P_app_ and absorption rate constant (*K*_a_) of monotropein and deacetylasperulosidic acid at pH = 7.4 were higher than those at pH = 6.8 and 5.4. In addition, the self-inhibition phenomenon depended on the perfusion concentration, suggesting that the dosage of the drug was a vital factor influencing self-absorption. Moreover, the *K*_a_ of monotropein was jejunum > duodenum > ileum, and that of deacetylasperulosidic acid was ileum > duodenum > jejunum. In the same intestinal segment, the *K*_a_ of monotropein was less than that of deacetylasperulosidic acid. In summary, the monotropein and deacetylasperulosidic acid could be well absorbed in different intestinal segments referring to P_app_ > 0.2 × 10^−4^ cm/s (Fagerholm et al., 1996; Hua et al., 2021). The primary absorption sites for monotropein and deacetylasperulosidic acid were jejunum and ileum, respectively.

The pharmacokinetic properties of monotropein orally administered at different doses (10, 20, and 40 mg/kg) in rats were determined accurately. Thus, plasma concentration-time curves suggested that monotropein was absorbed rapidly, in which the time reaching the peak concentration (T_max_) ranged from 1.3 to 1.4 h, and the half-time of elimination (T_1/2_) ranged from 2.9 to 4.5 h among the three gradient doses, and the maximum plasma concentration (C_max_) of monotropein was about 11.2–327.6 ng/mL. The C_max_ and the area under the curve (AUC_0-t_) appeared linear over the dose range of 10–40 mg/kg, which was in accordance with linear dynamic characteristics (Zhou et al., 2018).

Notably, the pharmacokinetic behavior is easily influenced by gender, disease state, the complexity of multiple components system, and processing of Chinese medicine (Franconi and Campesi, 2017; Zhang et al., 2019; Cao et al., 2021; Guo et al., 2022). The pharmacokinetic parameters of monotropein differed markedly between male and female rats. The C_max_, AUC_0-t_, and bioavailability of monotropein were higher in female rats than in male rats, but the apparent volume of distribution (V_d_) and clearance (CL) of monotropein were lower in female rats, indicating that monotropein was cleared more slowly in female than in male rats. For the complex chemical system, the perturbation from other co-existing components could not be ignored. After administration of major iridoids (50 mg/kg) and ethanol extracts (1,650 mg/kg) of *M. officinalis* How. (equivalent to 20 mg/kg monotropein), the C_max_, T_1/2_, AUC_0-t_, and AUC_0-∞_ of monotropein in the rats treated with major iridoid glycosides were higher than those in the rats treated with monotropein, while the T_max_ was shortened in the rats treated with major iridoid glycosides. This finding implied that monotropein had high bioavailability with facilitated absorption, increased exposure, and prolonged residence time in similar substances with major iridoid glycosides. In addition, the C_max_, AUC_0-t_, AUC_0-∞_, and absolute bioavailability of monotropein in the rats treated with ethanol extracts were lower than those in rats treated with major iridoid glycosides. The V_d_ and CL of monotropein in the treatment of ethanol extracts were much higher than those in the treatment of major iridoid glycosides, implying that monotropein was eliminated and distributed in the tissue rapidly under a complex chemical system with co-existing multi-components in the ethanol extracts from *M. officinalis* How. (Table 2) (Shen et al., 2018). Interestingly, the different enrichment methods for major iridoid glycosides with mutative elution conditions and filling materials or preparation methods for *M. officinalis* How. Guided by traditional Chinese theory might lead to different (even opposite) trends in the pharmacokinetic behavior (Li et al., 2016; Shi et al., 2017).

**TABLE 2:** Pharmacokinetic parameters of monotropein following oral administration.

Parameters	Units	Chemical system			
		Monotropein	Major iridoids of MO	Major iridoids of MO	Ethanol extracts of MO
C_max_	ng/mL	188.9 ± 37.9	451 ± 35	213.28 ± 36.72	265 ± 82
T_max_	H	1.3 ± 0.4	0.92 ± 0.14	2.08 ± 0.74	1.17 ± 0.29
T_1/2_	H	2.9 ± 1.6	7.11 ± 2.18	1.66 ± 0.45	3.00 ± 0.45
AUC_0-t_	ng·h/mL	1,114.0 ± 599.1	1,505 ± 189	1,014.13 ± 286.61	875 ± 182
AUC_0-∞_	ng·h/mL	1,217.8 ± 603.3	1,585 ± 279	1,370.15 ± 285.71	877 ± 182
AUMC_0-∞_	ng·h/mL	NA	8,546 ± 5,804	NA	3,064 ± 882
MRT	H	NA	5.17 ± 2.58	3.99 ± 0.83	3.47 ± 0.43
CL	L/g·h	NA	0.032 ± 0.006	0.018 ± 0.007	8.51 ± 2.61
V_d_	L/g	NA	0.32 ± 0.04	0.043 ± 0.021	1.932 ± 0.361
CL/F	L/g·h	0.022 ± 0.015	NA	NA	NA
V_d_/F	L/g	0.10 ± 0.032	NA	NA	NA
F	%	NA	3.69 ± 0.65	NA	2.04 ± 0.42
Dosage	mg/kg	20	50	20,000	1,650
Equivalent dosage for monotropein	mg/kg	20	19.3	16.8	20.95
Gender	NA	NA	Male rats	Male rats	Male rats
Administration style	NA	p.o	p.o	p.o	p.o
References	NA	Zhou et al. (2018)	Shen et al. (2018)	Li et al. (2016)	Shen et al. (2018)

C_max_, maximum plasma concentration; T_1/2_, elimination half-life; T_max_, time to reach C_max_; AUC, area under the concentration-time curve; AUMC, area under the first moment curve; MRT, mean residence time; V_d_, volume of distribution; CL, clearance; F, absolute bioavailability. MO, *Morindae officinalis* radix; NA, not available.

We showed that the tissue distribution of monotropein could be rapid and wide in the order of kidney > stomach > small intestine > liver > heart > lung > spleen and was eliminated rapidly without long-term accumulation in the above tissues, following oral administration at 20 mg/kg dose in rats. The content of monotropein was much lower in the spleen than in other tissues. The high content in the kidney indicated that the majority of monotropein was prone to elimination in the kidney (Zhou et al., 2018). Correspondingly, monotropein was widely distributed in the tissues or organs, including small and large intestine, spleen, stomach, liver, kidney, lung, thymus, heart, hypothalamus, marrow, ovary, testis, and uterus after oral administration of iridoid glycosides (equivalent to 40 mg/kg monotropein) at 100 mg/kg major in the rats of both sexes. Monotropein could be quantified using ultra-high performance liquid chromatography-tandem mass spectrometry, with optimized fragmentation transition as 408 → 211. Specifically, monotropein was widely distributed into the extravascular system of male or female rats. About 12 and 24 h post-oral administration, monotropein became undetectable. The highest content of monotropein was detected in the stomach and intestine, followed by spleen, kidney, heart, and testis at 1, 2, and 24 h in male rats. The highest content of monotropein in the female rats was observed in the uterus, ovary, marrow, hypothalamus, and liver at 0.5 and 1 h. In addition to the intestine and stomach, monotropein was present at a higher concentration in the spleen and heart of the male rats, while the content of monotropein in the liver, marrow, and hypothalamus in female rats was higher than that in male rats (Shen et al., 2018). The investigation of pharmacokinetics provided valuable information for developing monotropein or medical products containing monotropein.

Strikingly, gut microbiota also plays a crucial role in drug metabolism. Although there are few direct reports on the microbiota metabolism of monotropein, its metabolic behavior could be implied by the microbiota metabolism rule of some typical iridoids. The metabolites and metabolic pathways of five representative iridoids, including catalpol, geniposide, asperuloside, gentiopicroside, and morroniside, were investigated in the intestinal bacteria from adriamycin-induced nephropathic rats. After incubation for 24 h, the response values of aglycones of geniposide and morroniside were high, whereas the prototypes and aglycones of catalpol, asperuloside, and gentiopicroside were low. Nitrogenous metabolites of geniposide, asperuloside, gentiopicroside, and morroniside could be detected, and the content was related to the incubation time. In addition, the five iridoids could be easily metabolized by rat intestinal bacteria through phase I metabolic reactions, including hydrolysis, oxidation, and methylation reaction, whereas no phase Ⅱ metabolites were detected in anaerobic incubation system *in vitro* (Li et al., 2019; Shi et al., 2022). This finding implied that monotropein has similar metabolic behavior to these five representative iridoids, due to their similar chemical structure.

### 2.5 Pharmacological effects

#### 2.5.1 Improvement of osteoporosis

Osteoporosis is a systemic metabolic disease in the bone characterized by a decrease in bone mass and bone strength, increasing the risk of bone fracture. It is clinically manifested as a decrease in bone formation, an increase in bone resorption, loss of bone mass and quality, and deterioration of bone microarchitecture (Lupsa and Insogna, 2015). In terms of postmenopausal osteoporosis, estrogen deficiency is a leading cause of bone loss, which directly accelerates the differentiation of osteoclasts, promotes apoptosis in bone cells, and suppresses the activity of osteoblasts. Importantly, macrophage colony-stimulating factor (M-CSF) secreted from bone cells and osteoblasts, receptor activator of nuclear factor-κB ligand (RANKL), and the content of osteoprotegerin (OPG) are increased when estrogen is deficient, which indirectly intensifies the differentiation of osteoclasts (McNamara, 2010). Due to the negative effects of estrogen deficiency on bone, immunity could be affected in postmenopausal individuals, which might induce bone destruction, displaying a chronic inflammatory phenotype with abnormal cytokine expression and disturbed immune cell profile. These immune cells interact with osteoclasts and osteoblasts *via* cell-cell contact or paracrine, influencing the bone cells *via* OPG/RANKL, interleukin-6 (IL-6), and tumor necrosis factor-alpha (TNF-α). For example, specific subtypes of T lymphocytes secrete TNF-α, which induces osteoblast apoptosis and osteoclastogenesis *via* RANKL produced by B cells, triggering bone loss. Th-17 cells generate IL-17, which induces the differentiation of mesenchymal stem cells towards the osteogenic lineage and promotes the differentiation of osteoclasts. B lymphocytes regulate osteoclast formation by releasing granulocyte colony-stimulating factor and OPG/RANKL in estrogen deficiency. Moreover, neutrophils play a crucial role in bone homeostasis, of which over-activation under the condition of estrogen deficiency triggers osteoblast apoptosis by releasing reactive oxygen species (ROS) and increasing osteoclastogenesis *via* RANKL signaling pathway. Furthermore, mast cells might be associated with the development of postmenopausal osteoporosis due to the high storage of osteoclastic mediators (such as RANKL and IL-6) in their granules and the high cell number in osteoporotic bone (Sindhu et al., 2020; Fischer and Haffner-Luntzer, 2022).

Notably, monotropein has prospects of intervening in primary osteoporosis, including postmenopausal and senile osteoporosis. The number of osteoclasts differentiated from RAW264.7 cells induced by RANKL could be significantly decreased by monotropein. And monotropein inhibited osteoclast formation by decreasing the generation of nuclear factor of activated T cells and cytoplasmic 1 (NFATC1) *in vitro,* which is the signaling transduction terminal target for RANKL and plays an essential role in modulating the activity of osteoclasts (Song et al., 2019; Wang and He, 2020). In addition, the decreased activity of osteoblasts resulting from hydrogen peroxide (H_2_O_2_) could be reversed by monotropein at 0.001, 0.005, and 0.01 μg/mL *in vitro,* in which monotropein decreased TNF-α, IL-1, IL-6, and ROS levels and increased the content of alkaline phosphatase (ALP), matrix metalloproteinases (MMP), and M-CSF. In addition, it downregulated the protein levels of casepase-3, casepase-9, cyclooxygenase-2 (COX-2), inducible nitric oxide synthase (iNOS), and nuclear factor-kappa B (NF-κB) p65 and upregulated the protein expression of silent information regulator 1 (SIRT1). This finding suggested that monotropein improved the osteoblast capacity of anti-oxidant, proliferation, differentiation, and ossification by regulating the NF-κB signaling pathway and inhibiting inflammatory cytokine production (Zhu et al., 2016; Zhu et al., 2019). Intriguingly, the proliferation of preosteoblast MC3T3-E1 was increased when exposed to monotropein at 10, 25, 50, and 100 μM. The activity of ALP was significantly increased after incubation with monotropein for 72 h, and mineralization of MC3T3-E1 cells after incubation with monotropein for 28 days was increased, as assessed by alizarin red staining (Zhang et al., 2016).

The treatment of monotropein (40 or 80 mg/kg/day) for four successive weeks exhibited a protective effect on the ovariectomy-induced osteoporosis in mice, as indicated by the augmentation of mineral content and density in bone and improvement of bone microstructure, including bone volume fraction (for instance, the volume ratio of bone to tissue), structure model index, the bone surface to bone volume, trabecular thickness, trabecular separation and number, and connectivity density. Moreover, monotropein enhanced the elastic modulus of the femur and maximum load and stress and reduced the serum levels of IL-1, IL-6, and soluble RANKL (Zhang et al., 2016). Except for estrogen deficiency, inflammation also contributes greatly to bone metabolism and the occurrence of osteoporosis (Loi et al., 2016). This finding suggested that monotropein markedly improved bone microarchitecture and suppressed bone mass reduction by reducing the release of inflammatory cytokines and enhancing bone formation in lipopolysaccharide (LPS) and ovariectomy-induced osteoporotic mice. Moreover, monotropein could promote the activity and proliferation of ALP, mineralization of bone matrix, and the generation of osteopontin in osteoblastic MC3T3-E1 cells exposed to LPS. Moreover, monotropein reduced the generation of IL-6 and IL-1β, suppressed the nuclear translocation of NF-κB p65 and p50, and downregulated the phosphorylation of p65 and NF-κB inhibitor kinase, suggesting that monotropein could alleviate inflammation *via* the inactivation of NF-κB signaling pathway (He et al., 2018). Thus, monotropein can be served as promising medicine for anti-osteoporosis.

#### 2.5.2 Anti-oxidative effects

Postmenopausal and senile osteoporosis is induced by the accumulation of ROS (Davalli et al., 2016). Because of estrogen deficiency and aging, senile and postmenopausal females have excessive production and accumulation of ROS in a higher oxidative stress status, leading to impaired bone microstructure and osteoporosis (Mohamad et al., 2020). Oxidative stress can damage the cellular components in osteoblasts, which is an essential initiating factor to impair bone formation in postmenopausal osteoporosis (Baek et al., 2010; Cervellati et al., 2013). Dysfunction of the anti-oxidants causes a redox imbalance, resulting in a state of peroxidation and difficulty in the removal of ROS. The three classes of anti-oxidant defense systems eliminating peroxides and free radicals include anti-oxidant substances (glutathione, vitamin C, vitamin E, melatonin, ferritin, and ceruloplasmin), anti-oxidant enzymes (superoxide oxidoreductase, catalase, and glutathioneperoxidase), and repair enzymes (glycosylase, AP-endonuclease, DNA polymerase for DNA repair, phospholipase A2, and acyltransferase for lipid peroxide metabolism). Glutathione/oxidized glutathione (GSH/GSSG) conversion involves the phosphatidylinositol 3-kinase/protein kinase B-nuclear factor E2-related factor 2/heme oxygenase-1 (PI3K/Akt-Nrf2/HO-1) signaling pathway, and the anti-oxidant enzyme-mediated mitochondrial apoptosis pathway in osteoblasts is essential for the development of postmenopausal osteoporosis (Yang et al., 2022). Typically, oxidative stress is a key process of uncoupling between osteoblasts and osteoclasts in osteoporosis diseases (Kimball et al., 2021). The Nrf2/HO-1 signaling pathway is central in producing endogenous anti-oxidant enzymes. As a response to oxidative damage, Nrf2 is translocated into the nucleus, wherein it modulates the downstream anti-oxidant HO-1, which antagonizes ROS-induced oxidative stress (Waza et al., 2018; Zu et al., 2018). In addition, chloroquine, an autophagy inhibitor, mitigates bone loss and inhibits osteoclastic activity in ovariectomized mice (Florencio-Silva et al., 2018). Strikingly, autophagy is crucial in the pathophysiology of bone metabolism and cell cycle, including cell differentiation and function, with a critical role in osteoporosis (Yin et al., 2019; Li et al., 2020). Intriguingly, autophagy in bone cell is inversely related to bone loss and oxidative stress status in rats with estrogen deficiency induced by ovariectomy, and hence, the treatment targeting oxidative stress and autophagy could be a feasible approach in clinical setting (Yang et al., 2014; Ezzat et al., 2018).

H_2_O_2_ is a stable ROS that can diffuse through cell membranes and induce the generation of O_2_^−^ by activating nicotinamide adenine dinucleotide phosphate oxidase. Monotropein suppresses the H_2_O_2_-evoked ROS production in osteoblasts, which induces autophagy and exerts a protective effect on osteoblasts from cytotoxicity of H_2_O_2_. Moreover, it markedly ameliorates H_2_O_2_-induced oxidative stress by reducing the expression of malondialdehyde (MDA), superoxide dismutase (SOD), and catalase. Remarkably, monotropein downregulates the phosphorylation of Akt, mammalian target of rapamycin (mTOR), and the two downstream proteins (4EBP1 and p70S6K). The treatment of monotropein enhances autophagy in osteoblasts by upregulating the LC3-II/LC3-I ratio and Beclin1 expression. However, the effect of monotropein could be suppressed by the mTOR activator MHY1485 and Akt activator SC79. Consequently, the anti-oxidant effect of monotropein was partially modulated by boosting autophagy *via* Akt/mTOR signaling pathway (Shi et al., 2020).

Moreover, oxidative stress induces cellular injury and endothelial dysfunction, which mainly contributes to aging diseases. Monotropein enhanced the viability of H_2_O_2_-induced human umbilical vein endothelial cells (HUVECs). H_2_O_2_-mediated upregulation of proinflammatory cytokines, MDA, and endothelial cell adhesion factors were reduced by monotropein, while it reversed H_2_O_2_-induced downregulation of glutathione peroxidase (GSH-Px) and SOD activities. Furthermore, monotropein inhibited cellular apoptosis, NF-κB activation, and activator protein one expression in H_2_O_2_-stimulated HUVECs. However, the anti-apoptotic, anti-oxidative, and anti-inflammatory effects of monotropein could be counteracted by NF-κB activator. Accumulating evidence indicated that monotropein attenuated H_2_O_2_-induced oxidative stress and inflammation by regulating NF-κB/activator protein 1 (Jiang et al., 2020). The anti-oxidant capacities of monotropein against H_2_O_2_-induced oxidative stress cytotoxicity were investigated in PC12 cells, with IC_50_ value of 6.13 μg/mL (Yang et al., 2017).

Interestingly, monotropein could greatly ameliorate nephrotoxicity and decrease the generation of serum creatinine and blood urea nitrogen in mice with cisplatin-induced acute kidney injury (AKI). It also inhibited cisplatin-induced oxidative stress by increasing the activities of SOD, GSH, and catalase and decreasing MDA expression. The alleviation of cisplatin-induced AKI by monotropein was correlated with the activation of Nrf2/HO-1 signaling pathway, the inhibition of NF-κB signaling, and the regulation of apoptosis. These results suggested that monotropein could apparently alleviate cisplatin-induced AKI as a potential agent to attenuate the side effect of cisplatin (Zhang Y. et al., 2020).

Angiogenesis is essential for wound healing, especially that caused by chronic and ischemic injuries. New generated blood vessels are crucial for tissue recovery because they can provide cells at the wound site with oxygen and nutrition. The alleviation of oxidative stress-induced impairment and the promotion of differentiation of endothelial progenitor cells (EPCs) is essential for relieving ischemic injuries. Notably, EPCs are mononuclear progenitor cells in the bone marrow that can differentiate into endothelial lineage cells with vasculogenic effects (Asahara and Kawamoto, 2004). Monotropein significantly promoted the migration and tube formation of EPCs derived from bone marrow and prevented programmed cell death induced by tert-butyl hydroperoxide *via* apoptosis and autophagy by decreasing intracellular ROS generation and restoring mitochondrial membrane potential. These phenomena might be regulated by mTOR/p70S6K/4EBP1 signaling pathway and adenosine monophosphate-activated protein kinase (AMPK) phosphorylation. Moreover, wound healing was accelerated by monotropein in rats, as suggested by decreased healing time, decreased macrophage infiltration, and increased blood vessel formation. Overall, monotropein could improve wound healing *in vivo* by promoting differentiation and mobilization of bone marrow-derived EPCs and protecting against apoptosis and autophagy *via* inhibiting the AMPK/mTOR pathway; this observation deemed that monotropein is a valuable agent for wounds related to endothelial injury (Wang et al., 2018). In a molecular compatibility study, monotropein could synergistically exert antioxidant activity. MAS (monotropein, astragalin, and spiraeoside) promoted spermatogenesis in rats induced by varicocele by modulating the expression of cytokine and sex hormones and decreasing oxidative stress, endoplasmic reticulum stress, and apoptosis (Karna et al., 2019). Together, these studies suggested that monotropein is valuable in the prevention and treatment of oxidative stress-related diseases.

#### 2.5.3 Anti-nociceptive and anti-inflammatory effects

Previous studies showed that the stretching episodes and prolonged action time in mice and acute paw edema in rats could be significantly reduced after oral pre-treatment of monotropein at 20 and 30 mg/kg/day through a classic hot plate and writhing anti-nociceptive model or carrageenan-induced anti-inflammatory model, suggesting its potential anti-inflammatory and anti-nociceptive values (Choi et al., 2005). In addition, monotropein strongly inhibited the generation of nitric oxide and TNF-α. Desacetylasperuloside acid had no obvious activity against nitric oxide but had a specific TNF-α inhibitory activity (Shi et al., 2021), which revealed that the hydroxyl group at C-6 could decrease the activity, and the esterification of carboxyl group could enhance the activity (Recio et al., 1994; Viljoen et al., 2012). Furthermore, the major iridoid glycosides from *M. officinalis* had similar biological activity, enriched by XDA-1 macroporous resin, and the content of monotropein and deacetylasperulosidic acid was 35.9% and 25.9%, respectively. The major iridoid glycosides from *M. officinalis* significantly reduced the twisting frequency caused by acetic acid in mice and greatly decreased the air pouch granuloma in mice and the cotton pellet granuloma weight in rats at 50, 100, and 200 mg/kg. And these compounds also could significantly alleviate the paw swelling and decrease the weight loss, arthritic score, spleen index, and the serum content of IL-1β, IL-6, and IL-17a in complete Freund’s adjuvant-induced arthritic rats. An *in vitro* study showed that major iridoid glycosides from *M. officinalis* had an inhibitory effect on the release of inflammatory cytokines and the expression of COX-2, iNOS, and proteins associated with NF-κB signaling pathways and mitogen-activated protein kinase (MAPK) in LPS-stimulated RAW264.7 macrophages (Zhang Q. et al., 2020).

Moreover, monotropein exerted a chondroprotective activity by decreasing the proinflammatory cytokines, including TNF-α, IL-1β, and prostaglandin E2, in the knee joint tissue of osteoarthritic rats. The anti-catabolic and anti-apoptotic effects of monotropein on chondrocytes of osteoarthritic rats exposed to IL-1β were explored *in vitro*. In IL-1β-stimulated chondrocytes, monotropein ameliorated apoptosis at 25, 50, and 100 μg/mL. As a response to monotropein treatment, the production of MMP-3 and MMP-13 was markedly reduced, and the release of collagen type II alpha1 was increased. Hence, the anti-catabolic and anti-apoptotic activity of monotropein in chondrocytes supported the possibility of its therapeutic role in osteoarthritis (OA) (Wang et al., 2014). Monotropein inhibited the generation of *COX-2*, *iNOS*, *IL-1β*, and *TNF-α* mRNA in LPS-induced RAW264.7 macrophages. Monotropein treatment reduced the DNA binding activity of NF-κB. Consistently, monotropein downregulated the phosphorylation and degradation of inhibitory κB-α and consequently, the translocation of NF-κB. Furthermore, monotropein decreased the disease activity index, the activity of myeloperoxidase, and the expression of proteins related to inflammation by NF-κB inactivation in the colitis model induced by dextran sodium sulfate (Shin et al., 2013). Secondary liver injury occurs in patients with inflammatory bowel disease (Restellini et al., 2017), and monotropein might potentially alleviate secondary liver injury due to chronic colitis. It alleviated hepatic pathological damage caused by dextran sodium sulfate, infiltration of macrophages, liver parameters, and cytokine generations. The underlying mechanism of attenuating liver injury was closely related to the inactivation of the toll-like receptor 4 (TLR4)/NF-κB signaling pathway and downregulation of NLRP3 (NOD-, LRR-, and pyrin domain-containing protein 3) inflammasome (Chen et al., 2020). Taken together, monotropein inhibits the production of inflammatory mediators *via* NF-κB inactivation to intervene in colitis and its secondary disease. In summary, monotropein has a good performance in anti-nociceptive, anti-arthritic, and anti-inflammatory activities *via* NF-κB and MAPK signaling pathways.

#### 2.5.4 Treatment of muscle atrophy

**FIGURE 3:**
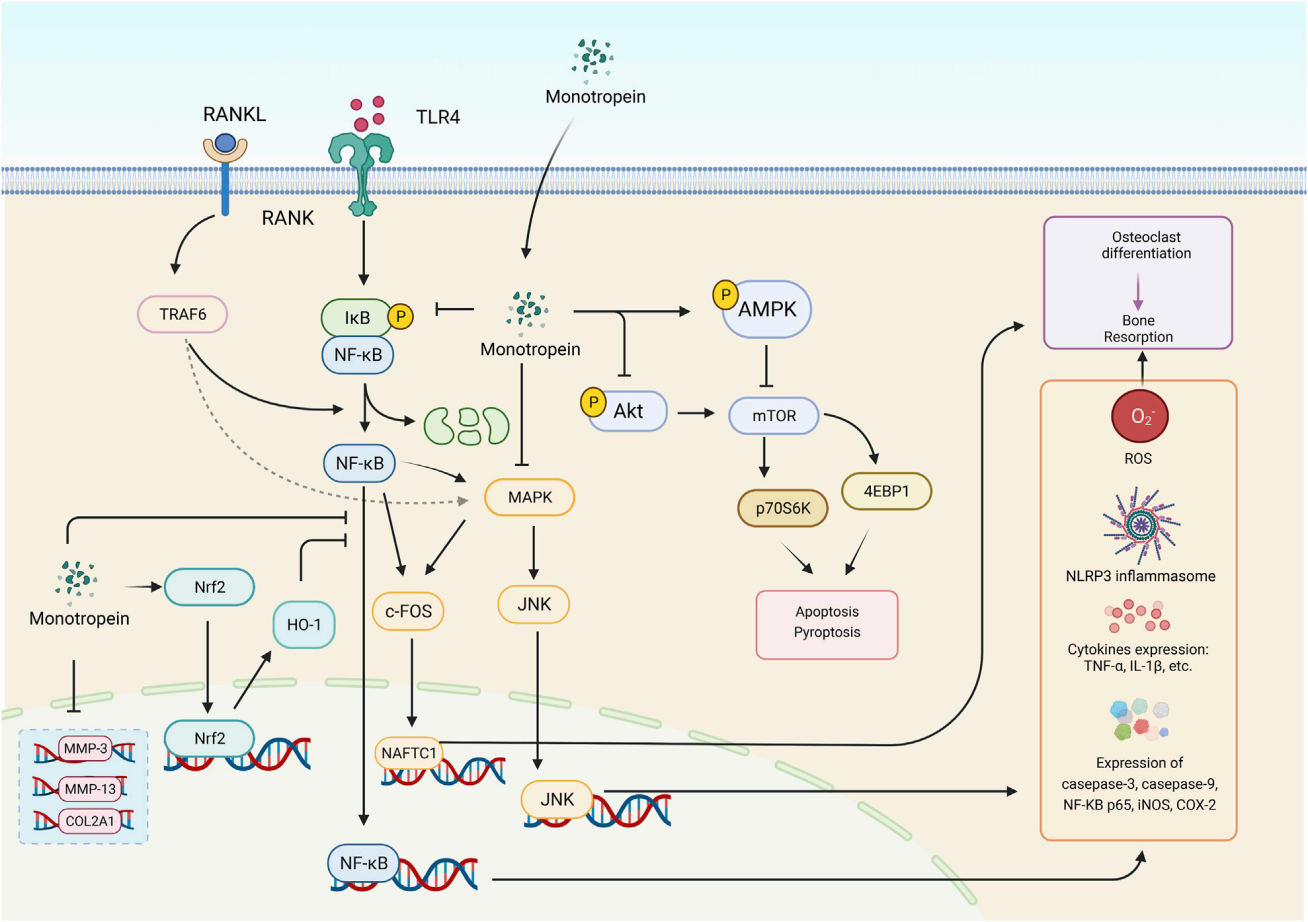
The various bioactivities of monotropein are associated with multiple signaling pathways. The crucial signaling pathways were displayed above, including NF-κB signaling pathway, PI3K-Akt signaling pathway, MAPK signaling pathway (depending on JNK signaling pathway), Nrf2/HO-1 signaling pathway and autophagy signaling pathway. RANKL, receptor activator of nuclear factor-κB ligand. RANK, receptor activator of nuclear factor-κB. TLR4, toll like receptor 4. MMP-3, matrix metalloproteinases 3. MMP-13, matrix metalloproteinases 13. Nrf2, nuclear factor E2-related factor 2. HO-1, Heme oxygenase-1. NFATC1, nuclear factor of activated T-cells, cytoplasmic 1. NF-κB, Nuclear factor-κB. MAPK, mitogen-activated protein kinase. Akt, protein kinase B. mTOR, mammalian target of rapamycin. p70S6K, p70 ribosomal protein S6 kinase. JNK, c-JunN-terminalkinase. 4EBP1, eukaryotic initiation factor 4E binding protein 1. ROS, reactive oxygen species. iNOS, inducible nitric oxide synthase. COX-2, cyclooxygenase-2. TNF-α, tumor necrosis factor-alpha. IL-1β, interleukin-1beta. c-FOS, c-FOS protein. COL2A1, collagen Type II alpha 1. TRAF6, TNF receptor-associated factor 6. IκB, inhibitor of NF-κB. NLRP3, nucleotide-binding oligomerization domain-like receptor family pyrin domain containing protein 3.

Importantly, monotropein improved muscle atrophy caused by dexamethasone by increasing muscle mass and strength in mice, which was associated with the increase in myosin heavy chain expression and the decrease in atrogin-1, muscle ring finger-1, and myostatin generation in the C2C12 myotubes and skeletal muscle in mice *via* activated Akt/mTOR/FOXO3a signaling pathway. Thus, monotropein is valuable in the prevention or treatment of muscle atrophy (Wang et al., 2022). The potential underlying mechanism of monotropein is outlined in Figure 3. Remarkably, the multiple bioactivities of monotropein, such as anti-osteoporosis, anti-oxidation, anti-inflammation, anti-nociception, and hepatic or renal protection, are closely associated with its capability of attenuating the activation of Nrf2/HO-1 signaling pathway, inhibiting MAPK signaling pathway, downregulating the NF-κB signaling pathway, and regulating the mTOR and autophagy signaling pathway.

#### 2.5.5 Pharmacological activity of deacetylasperulosidic acid

Importantly, deacetylasperulosidic acid, the metabolite of monotropein, exhibits various bioactivities, such as anti-oxidation, anti-inflammation, immune balance recovery, and improvement of obesity. For example, the treatment of deacetylasperulosidic acid decreased MDA and increased SOD activity in a dose-dependent manner (15, 30, and 60 mg/kg), with no influence on serum glutathione peroxidase activity, suggesting that deacetylasperulosidic acid could increase the catalase activity (Ma et al., 2013). In addition, deacetylasperulosidic acid inhibited the low-density lipoprotein-induced oxidation (63.8% ± 1.5%) at 20 μg/mL (Kim et al., 2005). In the case of anti-inflammation, deacetylasperulosidic acid suppressed the gene expression and secretion of cytokines and chemokines correlated with atopic dermatitis, such as TNF-α, IL-4, IL-1, IL-8, IL-6, IL-25, IL-33, macrophage-derived chemokines, thymic stromal lymphopoietin, thymus and activation-regulated chemokines, and monocyte chemoattractant protein-1 in HMC-1, HaCaT, and EOL-1 cells. Deacetylasperulosidic acid reduced histamine secretion in HMC-1 cells and controlled MAPK phosphorylation and the translocation of nuclear factor-kappa light chain enhancer of activated B cells into the nucleus by suppressing the decomposition of IκBα. Furthermore, deacetylasperulosidic acid stimulated the expression of proteins, such as involucrin and filaggrin in HaCaT cells, related to skin barrier functions. This finding confirmed that deacetylasperulosidic acid could mitigate atopic dermatitis by restoring immune balance and improving skin barrier function (Oh et al., 2021b). To recover immune balance, deacetylasperulosidic acid ameliorated pruritus and skin barrier dysfunction in atopic dermatitis induced by 2,4-dinitrochlorobenzene in NC/Nga mice (Oh et al., 2021a). Deacetylasperulosidic acid also inhibited the suppression of immunity mediated by cells with immunosuppressive substances isolated from freeze-dried ascites of Ehrlich carcinoma-bearing mice (EC-sup). In addition, it inhibited the decrease in IL-2 release in EC-sup-treated mice, while the natural killer cells could be activated in normal mice (Murata et al., 2014). For the improvement in obesity, deacetylasperulosidic acid could bind to peroxisome proliferator-activated receptor alpha with ligand efficiency similar to that of fenofirate and decreased proliferation of mature adipocytes, but not preadipocytes. The lactic dehydrogenase leakage was decreased by 43.03% after the treatment of deacetylasperulosidic acid in steatotic HepG2 cells at 50 μM. In the hyperlipidemic model induced by Triton WR-1339, 50 mg/kg deacetylasperulosidic acid remarkably lowered the triglyceride, total cholesterol, and low-density lipoprotein cholesterol levels by 40.27%, 46.00%, and 63.65%, respectively. In high-fat diet-fed animals, treatment with deacetylasperulosidic acid at 10 mg/kg decreased body mass index and ratio of abdominal circumference to thoracic circumference without influencing the specific rate of body mass gain. It also improved serum lipid, phosphatase, and transaminase contents in animals fed high-fat diet. The administration of deacetylasperulosidic acid lowered hepatocyte steatosis and adipocyte hypertrophy, suggesting its usefulness for the management of obesity (Esakkimuthu et al., 2019). Moreover, deacetylasperulosidic acid had a moderated activity for preventing the binding of SARS-CoV-2 spike protein to angiotensin-converting enzyme 2 for infection to host cells (West and Deng, 2021).

### 2.6 Toxicity

Although there are no studies on the toxicity of monotropein, some indirect evidence suggested that *M. officinalis* (the content of monotropein and deacetylasperulosidic acid were 35.9% and 25.9%, respectively) had no obvious toxicity at a maximum dose of 22.5 g/kg (Zhang Q. et al., 2020). It was also indicated that *M. officinalis* (the content of monotropein and deacetylasperulosidic acid were 38.6% and 23.6%, respectively) at the dose of 22.5 g/kg by oral administration did not cause any death in mice (Shen et al., 2018). Acute toxicity tests in mice demonstrated that ethanolic extracts of *M. officinalis* radix at a cumulative dosage of 250 g/kg/day also did not cause any death within 3 days (Zhang et al., 2000). However, direct evidence is required for the confirmation of the safe clinical application in the drug development of monotropein.

## 3 Discussion and conclusion

According to the theory of traditional Chinese medicine, *M. officinalis* radix enters the channel tropism of the liver and kidney and has the efficacy of tonifying kidney yang, dispelling wind and dampness, and strengthening tendons and bones with monotropein as the most abundant iridoid glycoside (Zhang et al., 2018; Kang et al., 2022). Consistently, monotropein is prone to accumulate in the kidney and liver, with a higher concentration in renal tissue, and is eliminated quickly with no long-term accumulation, deeming that the kidney and liver are vital target organs. Also, it was observed that most of the monotropein after oral administration was excreted through the kidney and liver. With a multitude of drug transporters and drug-metabolizing enzymes in the liver and kidney, the disposition of monotropein and its metabolites should be identified. The drug metabolizing enzymes mainly include cytochrome P450 enzyme, UDP-glucuronyltransferase, dihydropyrimidine dehydrogenases, sulfotransferases, and glutathione S-transferase, metabolizing monotropein *via* demethylation, oxidation, dehydrogenation, glucuronidation, sulfation, and glutathione (Kaur et al., 2020). The drug-metabolizing enzyme-mediated drug-drug interactions, including transport-transport, metabolism-metabolism, and transport-metabolism interplay, should be considered to assess the risks or benefits of drug combination with monotropein (Backman et al., 2016). Remarkably, this review proposed that monotropein had excellent anti-osteoporotic, anti-oxidative, anti-nociceptive, and anti-inflammatory bioactivities due to its ability of downregulating the NF-κB signaling pathway, inhibiting the MAPK signaling pathway, mitigating the activation of Nrf-2/HO-1 signaling pathways, and modulating the mTOR/autophagy signaling pathway. These pieces of evidence suggested that monotropein has a prospective value in drug development for osteoporosis and kidney diseases, especially in anti-osteoporosis.

Since postmenopausal osteoporosis is a multifaceted disease, the current therapeutic strategies of targeting osteocytes might not be sufficient. Thus, comprehensive approaches, such as improvement of systemic peroxidation state, chronic inflammatory stress, and immune status, as well as regulation of bone homeostasis, should be taken into consideration for treating postmenopausal osteoporosis. Also, disrupted gut microbial homeostasis, iron metabolism, cellular aging and stress, advanced glycation end products, and microRNA contributed to the pathogenesis of osteoporosis (Che et al., 2020; Yang et al., 2020; Chandra and Rajawat, 2021; Ge et al., 2022; Guan et al., 2022). Thus, the influence of monotropein on these biological processes should be explored.

Moreover, back pain is the most common symptom in patients with osteoporosis because of tension in muscular structures, joint imbalance, osteoporosis-related fractures, and skeletal deformities (Nagae et al., 2006; Iolascon et al., 2012), which also causes bodily changes (sensory, affective, and cognitive aspects). Acute pain is discovered in patients with fractures correlated with osteoporosis, wherein vertebral compression fractures experience high pain intensity and exert specific back pain (Sawicki et al., 2021). Severe osteoporosis can be characterized by chronic pain, mainly back pain. The pain in osteoporosis is related to osteoclast activity, central sensitization, neuropeptides, age-related raised density of sensory nerve fibers, and activation of the acid-sensing nociceptors (Catalano et al., 2017). The main reason is that high resorption in the bone can cause pathological changes in the bone sensory nerve fibers, with an overexpression of nociceptors sensitized by low pH environment. Overall, pain prevention is related to the optimal therapeutic strategy of osteoporosis, and multiple approaches are required for pain management in patients with osteoporosis to preserve and improve the quality of life (Julius and Basbaum, 2001; Mediati et al., 2014; Mattia et al., 2016). Therefore, monotropein has multiple advantages in managing pain and intervening etiologies of osteoporosis, based on its various pharmacological activities.

It was also demonstrated that iridoids could be metabolized by intestinal microbiota *via* hydrolysis, oxidation, reduction, and methylation reaction, with the generation of nitrogenous metabolites. Because of the high safety and rapid elimination, it is predicted that monotropein might undergo biological transformation *via* phase I reaction in intestinal microbiota, combined with hydrophilic endogenous molecules (phase II reaction) to form water-soluble metabolites, which will accelerate its excretion. Non-etheless, whether potential toxic metabolites, such as reactive dialdehyde intermediates, are produced during the metabolism by microbiota is yet to be elucidated.

Notably, monotropein converts slightly into deacetylasperulosidic acid during the extraction process, separation, and storage due to its structure instability, which poses significant challenges to the development and application of the drug (Wang et al., 2011). This necessitates a comparative study between monotropein and deacetylasperulosidic acid. In addition, the low bioavailability and fast elimination of monotropein caused by its strong polarity could not be ignored. Currently, few studies have been conducted to improve the chemical stability, total synthesis, structural modification, structure-activity correlations, bioavailability, and drug delivery systems, which would be helpful in improving the biological activity in drug development. Moreover, the approach to obtaining monotropein mainly depends on medicinal plant extraction, separation, and purification. Therefore, it is encouraging to develop the biosynthesis of this secondary metabolite, which is anticipated to be highly efficient, energy-saving, and environmental-friendly. More importantly, given the several bioactivities of monotropein, the clinical trial of monotropein is encouraged, especially in the treatment of osteoporosis and/or related complications, which would provide significant benefits to human health. In summary, this review provides a comprehensive overview of the basic information of monotropein, which could be valuable for related studies.
